# Decreased Circulating Melatonin with Loss of Age-Related Biphasic Change in Patients with Oral Squamous Cell Carcinoma

**DOI:** 10.3390/jpm11121357

**Published:** 2021-12-13

**Authors:** Yu-Fen Tsai, Yen-Yun Wang, Wan-Chi Tsai, Chang-Wei Su, Ching-Wei Hsu, Shyng-Shiou F. Yuan

**Affiliations:** 1 Graduate Institute of Medicine, College of Medicine, Kaohsiung Medical University, Kaohsiung 807, Taiwan; 970384kmuh@gmail.com; 2 Department of Hematology and Oncology, E-Da Cancer Hospital, Kaohsiung 824, Taiwan; 3 School of Chinese Medicine for Post Baccalaureate, College of Medicine, I-Shou University, Kaohsiung 824, Taiwan; 4 School of Dentistry, College of Dental Medicine, Kaohsiung Medical University, Kaohsiung 807, Taiwan; winnie30304@hotmail.com (Y.-Y.W.); grantkirros@gmail.com (C.-W.S.); jinkingtw@hotmail.com (C.-W.H.); 5 Center for Cancer Research, Kaohsiung Medical University, Kaohsiung 807, Taiwan; 6 Department of Medical Research, Kaohsiung Medical University Hospital, Kaohsiung 807, Taiwan; wanchi@kmu.edu.tw; 7 Department of Medical Laboratory Science and Biotechnology, College of Health Sciences, Kaohsiung Medical University, Kaohsiung 807, Taiwan; 8 Division of Oral and Maxillofacial Surgery, Kaohsiung Medical University Hospital, Kaohsiung 807, Taiwan; 9 Translational Research Center, Kaohsiung Medical University Hospital, Kaohsiung 807, Taiwan; 10 Department of Obstetrics and Gynecology, Kaohsiung Medical University Hospital, Kaohsiung 807, Taiwan

**Keywords:** oral cavity squamous cell carcinoma, melatonin, biomarker

## Abstract

Background: Melatonin, produced by the pineal gland, is known for its antioxidant, oncostatic, and anti-inflammatory properties. However, studies on serum melatonin levels in different cancer types have yielded conflicting results, and little is known about the clinical significance of serum melatonin in oral squamous cell carcinoma (OSCC) in the Southern Asian population. Therefore, we explored its role in OSCC in this study. Methods: A total of 67 male OSCC patients and 78 healthy controls were enrolled in this case–control study. The serum levels of melatonin were determined by enzyme-linked immunosorbent assay (ELISA) and compared between the two groups. Results: The serum melatonin levels were significantly lower in OSCC patients compared with healthy controls (mean ± standard deviation, 15.0 ± 4.6 vs. 18.5 ± 11.8 pg/mL, *p* = 0.02). In the subgroup of age less than 55 years (mean age of OSCC), OSCC patients had a significantly decreased melatonin level than healthy controls (mean melatonin, 15.7 ± 12.6 vs. 20.8 ± 3.9 pg/mL, *p* = 0.02). Decreased serum melatonin (odds ratio (OR): 0.95, 95%CI: 0.91–0.99), alcohol consumption (OR: 29.02, 95%CI: 11.68–72.16), betel quid chewing (OR:136.44, 95%CI: 39.17–475.27), and cigarette smoking (OR:29.48, 95%CI: 11.06–78.60) all increased the risk of OSCC under univariate analyses of logistic regression. Betel quid chewing (OR: 45.98, 95%CI: 10.34–204.49) and cigarette smoking (OR:6.94, 95%CI: 1.60–30.16) were the independent risk factors for OSCC in Taiwan. In addition, a negative correlation between age and melatonin level was observed in healthy controls (Pearson r = −0.24, *p* = 0.03). However, the negative correlation was lost in patients with OSCC. Melatonin concentration had no association with the severity of OSCC. Conclusion: Overall, our study provides evidence that serum melatonin levels decreased in OSCC patients in Taiwan and the decreased level is much significant in young populations and suggests that the decreased melatonin was associated with OSCC, especially in young populations. Further studies are warranted to investigate whether melatonin can be a useful non-invasive screening tool for OSCC.

## 1. Introduction

Oral cancer includes cancers of the lip, tongue, and oral cavity. Oral squamous cell carcinoma (OSCC) is the most common histological type of oral cancer. OSCC is associated with chronic exposure of cigarette smoking, alcohol, and betel quid chewing. OSCC usually occurs in males with the age from 60 to 80 years and is rare in young patients. OSCC in young patients is believed to be etiologically distinct from older patients [1,2,3]. However, the incidence among younger patients increased in recent years [4,5] and young patients with OSCC have a worse prognosis [5,6]. Therefore, early diagnosis of OSCC in young patients is critical.

While cigarette and alcohol consumption are the primary risk factors in Western countries, betel quid chewing and cigarette smoking are the leading causes in Taiwan [7,8]. In Taiwan, OSCC is the fourth highest incidence of malignancy in males and also ranks fourth highest cause of cancer death in man [9]. A retrospective study collected from Taiwan National Cancer Registry from 2002 to 2009 demonstrated that the five-year overall survival rate of oral cancer for stage I, II, III, and IV were 78.98, 69.38, 54.62, and 36.17%, respectively [10]. The disease is often diagnosed at an advanced stage III or IV and leads to poor prognosis [11]. All above, these facts highlight the value of early detection and diagnosis in OSCC. Routine examination of the oral cavity by dentists and other health care providers is the most widespread method for early detection of premalignant and malignant oral lesions. However, the prevalence of fear and anxiety for dental checkups is relatively high in adult populations, around 10 to 20% [12]. Therefore, an easily accessible biomarker for the early detection of OSCC is urgently need.

Melatonin is a hormone excreted by the pineal gland and is known to have antioxidant, oncostatic, and anti-inflammatory properties [13,14]. The role of melatonin in circadian rhythm is well documented and sleep deprivation has been associated with head and neck, breast, and prostate cancers [15,16]. However, studies on serum melatonin levels in different cancer types have yielded conflicting results. Some studies have shown that serum melatonin levels were higher in patients with multiple myeloma and melanoma [17,18], while others have found that they were decreased in patients with lung cancer, prostate cancer, and ovarian cancer [19,20,21].

A previous study reported that serum melatonin levels were decreased in OSCC patients in Romania and were associated with the severity of OSCC [22]. Therefore, the serum melatonin level may be a potential and easily accessible biomarker for early detection of the development of OSCC. However, the clinical significance of serum melatonin in OSCC patients in Taiwan is unclear. In this study, we explored the role of serum melatonin in OSCC patients in Taiwan.

## 2. Materials and Methods

### 2.1. Patient Enrollment

A total of 67 male OSCC patients diagnosed at Kaohsiung Medical University Hospital (KMUH) from January 2018 to December 2019 were included in the study. Since OSCC is highly prevalent in males than females, with a ratio ranging from 3:1 to 10:1 in Taiwan [23,24], we only included male subjects in our study. Newly diagnosed OSCC male patients more than 20 years of age were enrolled in this study. Patients with other malignancy or who had been exposed to chemotherapy or radiotherapy were excluded from the study. Clinical data were collected from the patients’ medical records. The disease staging was based on the 8th edition of the American Joint Cancer Committee Cancer Staging Manual [25]. Risk factors for OSCC, including alcohol consumption, betel quid chewing, and cigarette smoking, were also analyzed. None of the patients received any treatment prior to blood sample collection.

In addition, 78 age-matched individuals who received a general health check-up at KMUH were recruited as healthy controls. Controls with prior cancer history were excluded from the study. The study was approved by the Institutional Review Board of Kaohsiung Medical University Hospital, Kaohsiung, Taiwan (KMUHIRB-F(I)-20180069 and KMUHIRB-E(I)-20190009). Furthermore, written informed consent was obtained from each participant prior to the blood sample collection.

### 2.2. Sample Collection and Processing

Blood samples taken from OSCC patients and healthy controls were centrifuged and stored at −80 °C until enzyme-linked immunosorbent assay (ELISA) analysis. Serum melatonin was measured using a commercial kit (melatonin ELISA, RE54021, IBL-International, Germany). All procedures were performed according to the manufacturer’s instructions.

### 2.3. Statistical Analysis

Baseline characteristics between OSCC patients and healthy controls were analyzed using descriptive statistics and presented as frequencies, percentages, and means ± standard deviation (SD). Student’s *t*-test or nonparametric tests were used to test for statistically significant differences between continuous variables, whereas the chi-square or Fisher’s exact test was used for categorical variables. Pearson’s correlation was used to measure the correlation between the serum melatonin level and age. Logistic regression was used to determine risk factors for OSCC. With an α of 0.05, power of 0.80, and effective size of 0.5, the sample size was calculated by the G Power 3.1.9.7. [26], and the suggested sample size was at least 65 subjects in each group. Statistical significance was set at a two-tailed *p*-value smaller than 0.05. All statistical analyses were conducted using SPSS, version 22.0.

## 3. Results

### 3.1. Baseline Characteristics of Healthy Controls and OSCC Patients

A total of 78 healthy controls and 67 OSCC patients were enrolled in this study. The baseline characteristics are listed in Table 1. No differences were observed between the age of healthy controls and OSCC patients (*p* = 0.74), whereas significant differences were noted in the risk factors for oral cancer (alcohol consumption, betel quid chewing, and cigarette smoking) between these two groups (*p* < 0.0001). Specifically, more than three-quarters of OSCC patients had at least one risk factor exposure, whereas only approximately a quarter of healthy controls were exposed to alcohol consumption, betel quid chewing, or cigarette smoking. Among these OSCC patients, 26.8% were in early stages (stage I and II), 73.2% were in advanced stages (stage III, IVA and IVB), and 35.8% of the patients were positive for lymph node metastasis.

### 3.2. Serum Melatonin in Healthy Controls and OSCC Patients

**Figure 1:**
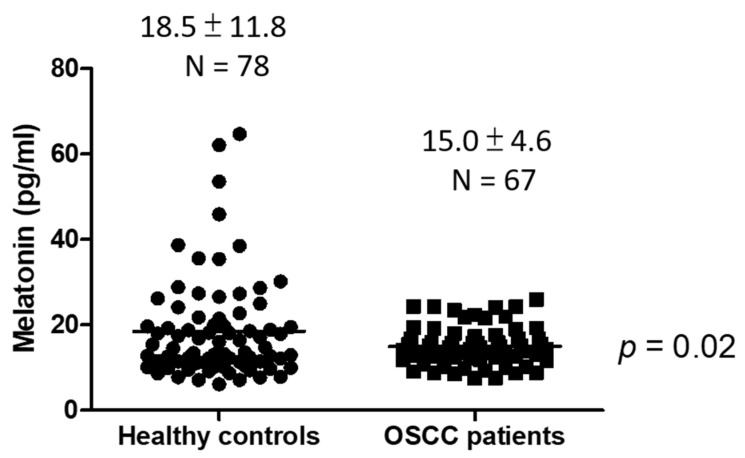
Serum melatonin levels in healthy controls and OSCC patients.

**Figure 2:**
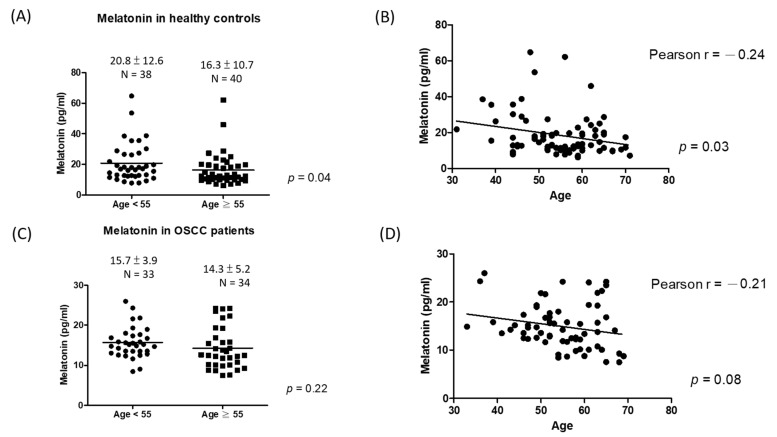
The correlation between age and melatonin levels in healthy controls and OSCC patients. (**A**) Melatonin levels in health controls stratified by the age of 55 years old; (**B**) the correlation between melatonin and age in healthy controls; (**C**) melatonin levels in OSCC patients stratified by the age of 55 years; (**D**) the correlation between melatonin and age in OSCC patients.

The serum melatonin levels were lower in OSCC patients than in healthy controls (mean ± SD, 15.0 ± 4.6 vs. 18.5 ± 11.8 pg/mL, *p* = 0.02, Figure 1). Among healthy controls, the serum melatonin levels were lower in older individuals (mean ± SD, 16.3 ± 10.7 pg/mL in age ≥ 55 years vs. 20.8 ± 12.6 pg/mL in age < 55 years; *p* = 0.04, Figure 2A), and a negative correlation between age and melatonin level was observed (Pearson’s r = −0.24, *p* = 0.03, Figure 2B). Of note, the negative correlation between the serum melatonin levels and age was lost in OSCC patients (Figure 2D). No significant difference in melatonin levels was observed between two age groups of OSCC patients (mean ± SD, 14.3 ± 5.2 pg/mL in age ≥ 55 years old group vs. 15.7 ± 3.9 pg/mL in age < 55 years old group; *p* = 0.22, Figure 2C).

### 3.3. Influence of Alcohol Consumption, Betel Quid Chewing, or Cigarette Smoking on Serum Melatonin Levels

In healthy controls, serum melatonin levels were similar in groups with or without exposure to alcohol drinking and cigarette smoking. Although the difference did not reach statistical significance, we did observe a trend of lower melatonin levels in healthy controls who chewed betel quid compared to those who did not (Table 2). Serum melatonin levels were similar in OSCC patients whether or not they consumed alcohol, betel quid, or cigarette. The detailed data are presented in Table 2.

### 3.4. The Correlation of Serum Melatonin with Surgical Staging, Tumor Size (T) and Lymph Node Metastasis (N) in OSCC Patients

**Figure 3:**
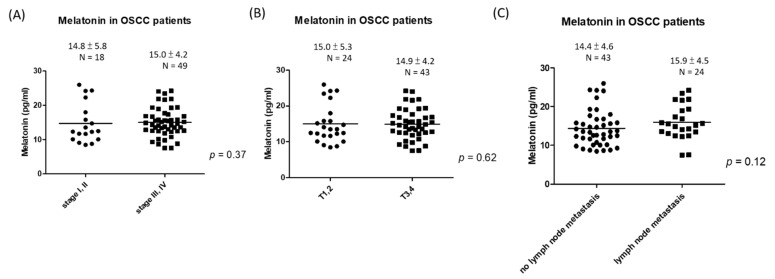
Serum melatonin levels in OSCC patients classified by (**A**) early stages (stage I, II) vs. advanced stages (stage III, IV). (**B**) T status: T1, T2 vs. T3, T4. (**C**) N status: negative vs. positive lymph node metastasis.

We further analyzed the association of serum melatonin with TNM staging. Serum melatonin levels were similar among different stages (early stages I and II vs. advanced stages III and IV, 14.8 ± 5.8 vs. 15.0 ± 4.2 pg/mL, *p* = 0.37, Figure 3A), tumor sizes (T1 and T2 group vs. T3 and T4 group, 15.0 ± 5.3 vs. 14.9 ± 4.2 pg/mL, *p* = 0.62, Figure 3B) and lymph node metastasis (negative vs. positive lymph node metastasis, 14.4 ± 4.6 vs. 15.9 ± 4.5 pg/mL, *p* = 0.12, Figure 3C).

### 3.5. Risk Factors for OSCC Patients in Taiwan

The risk factors for OSCC patients in Taiwan were listed in Table 3. In univariate analyses, decreased serum melatonin (odds ratio (OR): 0.95, 95%CI: 0.91–0.99), alcohol consumption (OR: 29.02, 95%CI: 11.68–72.16), betel quid chewing (OR:136.44, 95%CI: 39.17–475.27), and cigarette smoking (OR:29.48, 95%CI: 11.06–78.60) were all risk factors for the development of OSCC. After multivariate analyses, it was evident that betel quid chewing (OR: 45.98, 95%CI: 10.34–204.49) and cigarette smoking (OR:6.94, 95%CI: 1.60–30.16) were the two main risk factors for OSCC in Taiwan.

## 4. Discussion

In the present study, we observed that the serum melatonin levels were significantly lower in OSCC patients than in healthy controls. This result was consistent with the previous study conducted in Romania by Stanciu et al. Their data revealed that melatonin concentrations were significantly lower in OSCC patients than in controls (18.2 vs. 47.6 pg/mL, *p* < 0.001) [22]. In addition, as well as in OSCC, the serum melatonin levels have also been found to be lower in prostate cancer [20] and lung cancer [21]. All these findings suggest the oncostatic and tumor inhibitory effects of melatonin.

Stanciu et al. found that serum melatonin was inversely associated with the presence of lymph node metastases in Romanian OSCC patients [22]. In addition, they reported that the serum melatonin levels were negatively correlated with matrix metalloproteinase-9 (MMP 9). Low levels of serum melatonin and high levels of serum MMP-9 were correlated with larger tumor sizes and higher invasiveness and lymph node metastasis [27]. However, in our study, we did not observe a correlation between serum melatonin and tumor size or lymph node metastasis. This inconsistency may be attributed to the high incidence of betel quid chewing among Taiwanese patients with OSCC. Arecoline is a natural alkaloid in betel nut. Arecoline treatment (10 mg/kg body weight daily for 10 days) suppresses pineal activity and decreases serum melatonin levels in rats [28]. Moreover, another possible explanation for the discrepancy between the findings of Stanciu et al. and ours is ethnic difference. Previous studies have suggested that Asian populations produce lower melatonin levels than Caucasian, African American, and Ghanaian populations [29,30]. Taken together, betel quid chewing and ethnic difference may contribute to the result inconsistency in OSCC patients in Taiwan and in Romania.

Melatonin levels are known to gradually decline with age [31]. In our study, we found a negative correlation between melatonin levels and age in healthy controls, but not in OSCC patients. To the best of our knowledge, this is the first study to demonstrate the loss of negative correlation between age and melatonin in OSCC patients. In addition, in the subgroup of age less than 55 years (mean age of OSCC), OSCC patients had a significantly decreased melatonin level than healthy controls (mean melatonin 15.7 vs. 20.8 pg/mL) compared to the whole population (mean melatonin in OSCC patients 15.0 vs. healthy controls 18.5 pg/mL). From prior studies, the young OSCC patients seemed to have a higher rate of distant metastasis and a worse prognosis [5,6]. Earlier diagnosis of OSCC is necessary to improve the survival of OSCC, especially in young populations. Therefore, the serum melatonin level may have a role for the detection of OSCC in young populations.

Furthermore, we did observe a trend of lower melatonin levels in healthy controls who chewed betel quid compared to those who did not, though the difference did not reach statistical significance. The condition is compatible with the finding from an animal study which found that arecoline treatment suppresses pineal activity and decreases serum melatonin levels in rats [28]. In addition, we did not find any influence of alcohol, betel quid, and cigarette consumption on the serum melatonin levels in OSCC patients with OSCC. No good explanation for the difference in health controls and OSCC currently exists. Due to the small sample sizes in each group for the statistical analysis, it is difficult to draw conclusions about the influence of alcohol, betel quid, and cigarette consumption on serum melatonin levels in our study. More studies are needed to understand the influence of alcohol, betel quid, and cigarette consumption on the serum melatonin levels.

To determine the risk factors of OSCC in Taiwan, we found that serum melatonin, alcohol consumption, betel quid chewing, and cigarette smoking are all risk factors for OSCC in univariate analyses. However, after multivariate analyses, it was evident that betel quid chewing and cigarette smoking were the two main risk factors for OSCC in Taiwan. Serum melatonin was no longer the risk factor for OSCC and the betel quid had the highest OR. These results highlight the importance of betel quid in the carcinogenesis of OSCC in Taiwan and the different etiology of OSCC between Taiwan and Western countries. In our study, we could not find a significant influence of alcohol, betel quid, and cigarette consumption on serum melatonin. However, arecoline did suppress the pineal activity and decrease the serum melatonin levels in animal model [28]. Therefore, the interaction between betel quid and melatonin could not be excluded. Moreover, numerous factors, such as diet, medications, and sleep quality also influence the melatonin levels [32,33]. Thus, the decrease in melatonin levels observed in OSCC patients could possibly be the result of other factors. More-extensive and larger-scale studies are required to confirm the role of melatonin in the detection of OSCC.

There are two limitations for our study. First, the size of the study population was small; a total of 67 male OSCC patients and 78 healthy controls were enrolled in this study. Nonetheless, the sample size of our study was nearly double that of the study by Stanciu et al. [22]. Second, the blood samples used in this study were not collected at fixed time point, which may cause variations in the serum melatonin levels. Despite these potential limitations, this is the first study to evaluate the association of serum melatonin and OSCC patients in Asian populations, especially those with betel quid chewing as a specific risk factor, which is not a common exposure factor for the Western population.

## 5. Conclusions

The present study observed lower serum melatonin levels in OSCC patients in Taiwan and the decreased level was very significant in young populations. A negative correlation between age and melatonin level in healthy controls was observed and was not found in OSCC patients. More large-scale studies are warranted to confirm whether melatonin is applicable as a non-invasive screening tool for OSCC.

## Figures and Tables

**Table 1:** Baseline characteristics in healthy controls and OSCC patients.

	Healthy Controls (N = 78)	OSCC Patients (N = 67)	*p* Value
Age (mean ± SD)	54.5 ± 8.6	54.5 ± 8.3	0.74
Melatonin (mean ± SD, pg/mL)	18.5 ± 11.8	15.0 ± 4.6	0.02
Alcohol consumption (N)	9	53	<0.0001
Betel quid chewing (N)	4	59	<0.0001
Cigarette Smoking (N)	20	61	<0.0001
Stage			
I (N)		7	
II (N)		11	
III (N)		12	
IVA (N)		26	
IVB (N)		11	
Lymph node metastasis			
Yes (N)		24	
No (N)		43	

Abbreviations: OSCC: oral squamous cell carcinoma, SD: standard deviation.

**Table 2:** Serum melatonin levels classified by alcohol consumption, betel quid chewing, and cigarette smoking among healthy controls and OSCC patients.

		Healthy Controls (N = 78)			OSCC Patients (N = 67)		
		N	Melatonin (Mean ± SD)	*p* Value	N	Melatonin (Mean ± SD)	*p* Value
Alcohol consumption	Yes	9	18.9 ± 10.7	0.90	53	14.8 ± 4.3	0.55
	No	69	18.4 ± 12.0		14	15.6 ± 5.8	
Betel quid chewing	Yes	4	15.3 ± 10.0	0.58	59	14.9 ± 4.6	0.97
	No	74	18.7 ± 11.9		8	14.9 ± 5.2	
Cigarette smoking	Yes	20	17.5 ± 9.9	0.65	61	15.0 ± 4.8	0.78
	No	58	18.9 ± 12.4		6	14.4 ± 1.8	

Abbreviations: OSCC, oral squamous cell carcinoma; SD, standard deviation.

**Table 3:** Risk factors for OSCC patients in Taiwan.

	Univariate Analyses		Multivariate Analyses	
Variables	OR (95% CI)	*p*-Value	OR (95% CI)	*p*-Value
Age	1.00 (0.96–1.04)	0.98	0.94 (0.86–1.02)	0.14
Serum Melatonin	0.95 (0.91–0.99)	0.03	0.93 (0.85–1.03)	0.18
Alcohol consumption	29.02 (11.68–72.16)	<0.01	2.80 (0.65–11.97)	0.17
Betel quid chewing	136.44 (39.17–475.27)	<0.01	45.98 (10.34–204.49)	<0.01
Cigarette Smoking	29.48 (11.06–78.60)	<0.01	6.94 (1.60–30.16)	0.01

Abbreviations: OR: odds ratio.

## Data Availability

The data used in the present study are available from the corresponding author upon reasonable request.
